# A Two-Channel DFT Spectrum Analyzer for Fluctuation Enhanced Sensing Based on a PC Audio Board

**DOI:** 10.3390/s21134307

**Published:** 2021-06-24

**Authors:** Emanuele Cardillo, Graziella Scandurra, Gino Giusi, Carmine Ciofi

**Affiliations:** Department of Engineering, University of Messina, 98100 Messina, Italy; ecardillo@unime.it (E.C.); gino.giusi@unime.it (G.G.); carmine.ciofi@unime.it (C.C.)

**Keywords:** low-frequency noise measurements, signals elaboration, sound boards, spectrum analyzer

## Abstract

The main requirement for using the Fluctuation Enhanced Sensing technique is the ability to perform low-frequency noise measurements. The portability of the measurement system is also a quite desirable feature not limited to this specific application. In this paper, an approach for the realization of a dual channel spectrum analyzer that is capable of exploring frequencies down to DC, although based on a USB sound card, is proposed. The lower frequency range of the input signals, which is outside the frequency range of the sound board, is upconverted to higher frequencies by means of a very simple modulation board. Then, the entire spectrum is reconstructed numerically by proper elaboration. With the exception of the modulation board, the approach we propose does not rely on any specific hardware. Thanks to the efficiency of the spectra estimation and reconstruction software, which is based on a public domain library, the system can be built on a low-cost computer single board computer, such as the Raspberry PI3. Moreover, when equipped with an optical TCP/IP link, it behaves as a compact spectrum analyzer that along with the device under test can be placed into a shielded environment, thus being isolated from external electromagnetic interferences.

## 1. Introduction

The development of advanced sensing techniques requires the availability of dedicated instrumentation which, not being commercially available, must be custom developed. Among the advanced sensing techniques, the Fluctuation Enhanced Sensing (FES) technique shows great potential as it extends the range of information that can be extracted from a single sensor [1,2,3,4,5]. FES is based on the principle that the stochastic fluctuations of the sensor signal, due to the interactions at the microscopic level between the sensor surface and the agents to detect, are recorded and analyzed rather than its average value. As a result, a single sensor can act as a complex high-dimensional electronic nose or tongue and, as a consequence, FES can exhibit a sensitivity of orders of magnitude higher than that of conventional sensing systems. A few open questions must be addressed before FES can become commercially available: an FES system requires the sensor interface and measurement instrumentation to be dedicated and so specifically designed to extract and amplify the low-frequency stochastic signal components, which are usually orders of magnitude lower than the conventional deterministic sensor signals [2,6,7]. After extraction and amplification, proper selected statistical properties of the noise signals are analyzed to generate a corresponding “stochastic fingerprint” of the agent. The power spectral density of the noise signal is often used as such a fingerprint; therefore, an in-depth experience in the field of Low-Frequency Noise Measurements (LFNM) can be useful for FES application. In fact, LFNMs have long been used as one of the most sensitive tools for the investigation of the quality and reliability of electron devices [8,9,10,11,12,13]. When an electron device is DC-biased, currents or voltages fluctuate as a result of the interaction between the charge carriers and the detailed microstructure of the device. Most of these interactions result in flicker (or 1/*f*^γ^) noise components that are more easily detected at very low frequencies where their contribution overcomes the unavoidable thermal noise components [14]. In many cases, noise measurements have proved to be decisive in understanding the detailed conduction mechanisms of electron devices [15,16,17,18,19]. This represents an invaluable feature in the process of developing emerging device technologies, whereby the clear understanding of the microscopic behavior is a key factor for the design of the next-generation devices and, clearly, it was obvious to extend this potential to advanced sensing techniques, such as the FES technique. In almost all the above cases, the frequency range of interest extends well below the AC cut-in filter of conventional benchtop or board-based DFT spectra analyzers (a few Hz). Therefore, in any actual measurements, the DFT input stages are DC coupled to the high-gain preamplifier connected to the Device-Under-Test (DUT), which takes care of rejecting the DC components due to the DC bias, with a corner frequency that is often in the order of a few mHz. Although in principle, there is no reason not to explore even lower frequencies, this is rarely done due to the long measurement time required to obtain a reliable estimate of the spectrum at such low frequencies [14]. The fact that we are mostly interested in the frequency range from a few tens of mHz up to a few tens of Hz in FES applications rules out the possibility of directly using PC sound boards because their input is AC coupled with a cut-in frequency in the order of a few Hz. This is unfortunate, since modern PC sound cards (either integrated or as add-on boards) are available at very low cost and have otherwise quite remarkable characteristics, e.g., sampling frequencies often in excess of 100 kHz, a resolution of 24 bits, and quite good matching between the left and right channels. Moreover, they are intrinsically very well integrated as part of a personal computer that represents a very powerful digital signal processor, relying on a wide range of proprietary and/or public domain drivers and software tools. Overcoming the frequency limitations that prevent sound boards from being used as inexpensive and effective alternative to conventional benchtop and board-based spectral analyzers is indeed the subject of this work. As it will be shown in the next few sections, by means of a very simple add-on board composed by a low pass filter and an analog multiplier for each channel and by resorting to proper digital signal processing, it is possible to reach the goal of realizing a two-channel spectrum analyzer with a low-frequency corner virtually extending to DC and with overall performances that are completely satisfactory for performing FES and LFNM, even when using cross-correlation approaches.

## 2. Proposed Approach

**Figure 1:**
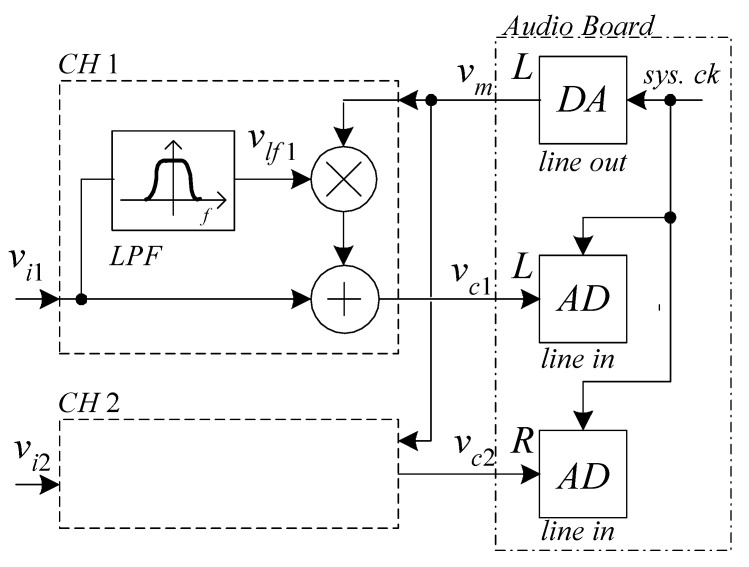
Block diagram illustrating the principle of operation of the proposed approach. The block indicated with *CH*2 is identical to the block *CH*1. The signals *v_i_*_1_ and *v_i_*_2_ come from the outputs of the low noise preamplifiers that are part of the low noise measurement set up. The system can be used in single or dual channel mode, or to estimate the cross spectrum between the inputs.

The approach we propose relies on the system configuration shown in Figure 1. The audio board generates a sinusoidal carrier (*v_m_*) used to translate the low-frequency portion of the input signals (*v_lfx_*) at a higher frequency within the pass bandwidth of the acquisition system.

The signal *v_c_*_1_ can be written as:

(1) $$v_{c1}\left(t\right)=v_{i1}\left(t\right)+k_{V}v_{lf1}\left(t\right)A_{m}\cos\left(2\pi f_{m}t\right)$$

where *A_m_* is the amplitude of the modulating signal *v_m_* whose frequency is *f_m_*, and *k_V_* is the multiplier scale factor. In order to simplify the discussion, we will assume *k_V_A_m_* = 1, which is indeed the amplitude factor employed in the proposed measurement setup. The signal *v_i_*_1_ typically comes from the output of a Low-Noise Amplifier (LNA) that rejects the DC component across the DUT and raises the level of the noise signal to be investigated so that the quantization error introduced by the converters inside the audio board can be minimized. The LNA is typically part of the low-noise measurement set up, and in many cases, custom designs must be used, depending on the characteristics of the DUT. In the case of the measurements that have been performed to test our system, we realized a rather simple LNA configuration, as it will be discussed in Section 4.

**Figure 2:**
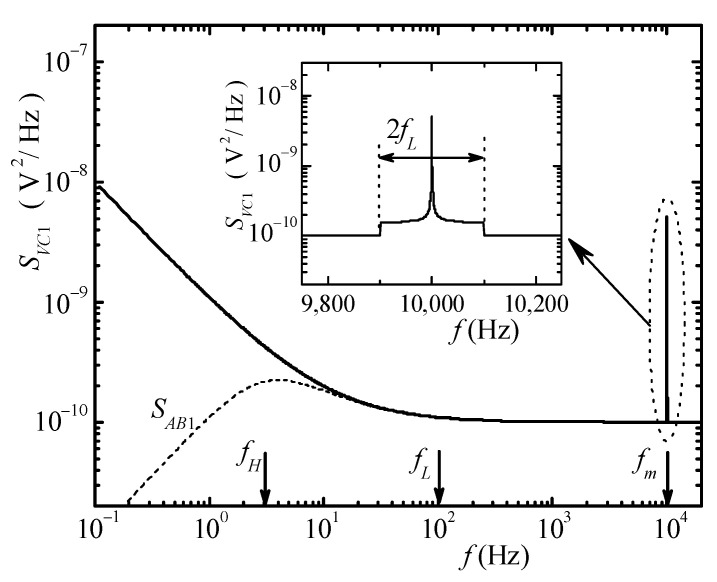
Calculated spectra illustrating the operation of the system. The black line represents the PSD of the signal vc1, while the dotted curve represents the spectra that would be obtained because of the high-pass filter at the input of the audio board. The inset represents the PSD close to the carrier frequency as the result of the operation in the front-end board.

A typical spectrum for *v_c_*_1_, as can be calculated starting from Equation (1), is represented in Figure 2, with the assumptions of ideal low-pass filter (gain 1 and bandwidth *f_L_* for LPF in Figure 1). The frequency *f_H_* in Figure 2 marks the typical low-frequency corner of the high-pass filter at the line input of the audio board that we assume to be part of the AD converter block in Figure 1.

The spectrum *S_vc_*_1_ of *v_c_*_1_ is the result of the superposition of the spectrum *S_vi_*_1_ of the input signal *v_i_*_1_ with the Double-Sided Band-Suppressed Carrier (DSB-SC) modulated low-frequency portion of the spectrum across the carrier frequency *f_m_*. By means of the DSB-SC modulation, the low-frequency portion of the spectrum is preserved notwithstanding the presence of the high-pass filter within the line input conditioning chain prior to the actual sampling in the audio board. Clearly, the low-frequency portion of the spectrum is now superimposed to the spectrum of the input signal at frequencies near *f_m_*, and therefore, we must ensure the possibility of retrieving the spectrum *S_VLF_*_1_ of *v_lf_*_1_ from the combined spectrum *S_Vc_*_1_.

In the case of LFNMs for FES, this can be obtained quite easily since (a) for LFNM to be of any use, the Power Spectral Density (PSD) at low frequencies has to be much larger than the PSD at higher frequencies (white noise region); (b) in almost all the LFNM experiments, a white noise region of the spectrum exists at higher frequencies whose PSD can be easily estimated and, therefore, subtracted from the spectrum in the region close to *f_m_*. In the system we devised, both the modulation amplitude *A_m_* and the frequency *f_m_* can be adjusted. In particular, the system can operate with *A_m_* = 0, thus allowing the user to select the most proper region (flat PSD) for positioning the modulated spectrum. It is worth noting that by using a logarithmic frequency scale, i.e., the most common frequency scale representation in LFNM, the portion of the high-frequency spectrum occupied by the modulated signal is very narrow in terms of fractional bandwidth. This means, for all intents and purposes, that the correct spectrum might be straightforwardly obtained by subtracting a constant value from the demodulated spectrum. In other words, assuming that the PSD *S_AB_* of the signal acquired by the audio board is known, the PSD *S_VLF_* of the low-frequency component of the input signal can be obtained as follows:

(2) $$S_{VLF}\left(f\right)=G_{D}\;\left[S_{AB}\left(f-f_{m}\right)-S_{AB}\left(f_{m}\right)\;\right]$$

where *G_D_* is a proper gain coefficient depending on the carrier amplitude and on the multiplier constant in Figure 1.

Although this procedure may appear quite simple, when it comes to the actual algorithms that can be used for PSD estimation, using Equation (2) is anything but straightforward. When dealing with sampled signals, the most common approach for PSD estimation is to resort to the modified periodogram approach [20] that relies on the properties of the Discrete Fourier Transform (DFT) for the estimation of the spectrum at discrete frequencies *f_k_*:

(3) $$f_{k}=k\Delta f;\;0\leq k<N;\;\Delta f=\frac{f_{S}}{N}$$

where *f_S_* is the sampling frequency and *N* is the number of samples in each record used for spectral estimation. The quantity Δ*f* in Equation (3) is the frequency resolution for spectral estimation. The estimation of the PSD in the modified periodogram approach is obtained by dividing the power of the signal in a bandwidth *NBW* (Noise Band-Width) centered at each *f_k_* and dividing such power by the *NBW*. As long as the actual PSD of the signal does not change too much within the NBW, we obtain a sufficiently accurate estimation of the PSD of the signal. Unfortunately, in the case of LFNM, the assumption of almost constant PSD within a given *NBW* is generally not applicable at the lowest frequencies *f_k_* in Equation (3). This means that if we are interested in the correct estimation down to a minimum frequency *f_min_* (say 100 mHz), the NBW must be conveniently smaller. This issue is discussed in detail in [21], where it is shown that since in the case of conventional PSD estimation, the NBW is about Δ*f*, one must always work with Δ*f* << *f_min_*. The very same arguments that apply in the case of direct flicker noise measurements also apply to the spectrum close to the carrier in our approach. This means that if we are interested in the PSD down to *f_min_* = 100 mHz (a quite typical value for LFNM), we must operate with Δ*f* in the order of 10 mHz or so. In the case of many benchtop spectrum analyzers, in order to obtain such low values of frequency resolutions without a too large value for the record length *N*, the input signal is low-pass filtered and sampled at a relatively low frequency. Unfortunately, we do not have this option, since the information about the low-frequency spectrum is across the carrier frequency. Even by using the lowest standard sampling frequency for an audio board (44.1 kHz), the record length required for obtaining Δ*f* = 10 mHz would be in the order of 5 × 10^6^ with a duration of 100 s for each record. Moreover, since several records need to be averaged to reduce the statistical error associated with the modified periodogram approach, this means that we would have to wait several minutes to obtain a reasonable estimate of the PSD at all frequencies. Therefore, this approach would be quite unpractical.

A second approach would be to perform a digital demodulation of the DSB-SC signal. In this way, we can estimate the PSD of *S_AB_* with a conveniently larger *Δf* for obtaining information on the spectrum of the input signal at higher frequencies in a matter of a few seconds with a low *N*; at the same time, the demodulated signal could be band limited and decimated so that a conveniently lower equivalent sampling frequency could be used for obtaining a narrow *Δf* while maintaining *N* in the order of a few thousands. The feasibility of this approach was explored on a single channel [22]. However, it requires that a coherent demodulator be numerically implemented and the carrier reconstruction from the sampled signal can be quite challenging, since the presence of a not negligible residual carrier amplitude must be ensured by acting on the front-end analog board.

A third approach that addresses and solves all the problems mentioned above is based on the use of the Quasi-Logarithmic Spectral Analyzer (QLSA) library [23] completed with the extension for Narrow Band Spectral Estimation (NBSE) discussed in [24]. In [23], we discussed the features of the public domain library QLSA. This library allows performing multi-channel spectra and cross-spectra estimation by dividing the frequency region of interest in equally sized (in a logarithmic scale) overlapping frequency regions. For each frequency region, the most proper resolution bandwidth is used for an optimal compromise between frequency resolution and measurement time. The library essentially behaves as a large number of spectral analyzers all working in parallel and performing PSD estimation with the same *N* but with different sampling frequencies and, hence, with different resolution bandwidths. The library has been expanded with the introduction of functions that, by taking advantage of the properties of the Chirp Z-transform [25], allow performing the estimation of the PSD in a narrow bandwidth with high frequency resolution and narrow NBW while using short records. The NBSE extension was mainly developed to allow for the measurement of strongly peaked noise spectra obtained in a circuit containing a quartz tuning fork used as a sensor [24]. This is a situation quite similar to the problem we have to face for the estimation, with high resolution, of the spectrum across the carrier frequency in Figure 2. As a matter of fact, by using QLSA and the NBSE extension, the direct implementation of Equation (2) is greatly simplified. Moreover, QLSA is quite optimized, and it allows all the operations required for spectral estimation (included carrier generation and cross spectra extraction) to be performed with low computation resources, even on small board computers. Therefore, the entire system can be regarded as an embedded spectrum analyzer that employs commercial hardware, with the exception of the rather simple board required for the analog signal elaboration in Figure 1. As we will discuss in the following, for testing the proposed approach, we developed a two-channel spectrum analyzer employing a PI3 Raspberry board computer, with an USB audio board for signal acquisition. The system behaves as an autonomous spectrum analyzer that can be networked for configuration and for monitoring the result of spectra estimation. To obtain the complete galvanic insulation from the external environment, a standard optical IP link can be optionally employed. Needless to say, with the approach we have followed and because of the very low cost required for its implementation, several spectrum analyzers can be realized and networked together for expanding the measurement capability while, at the same time, centralizing instrumentation management and data collection. In the following section, we will discuss in detail the hardware implementation of the front-end and discuss how QLSA can be used to easily extract all desired spectra information.

## 3. System Implementation

This section may be divided by subheadings. It should provide a concise and precise description of the experimental results, their interpretation, as well as the experimental conclusions that can be drawn.

As stated before, the approach we propose is of rather general application in the sense that it does not rely on a specific audio board or operating system for its operation. All software libraries, including QLSA, are available for Linux and Windows platforms, so that the actual system implementation may only differ depending on the specific application. For instance, for a fixed measuring station, when electromagnetic interferences are not a primary concern, a personal computer is likely to have already all the required hardware components (audio board and communication interface) with the exception of the front-end board. If we require a compact system with the ability to allow complete galvanic insulation, an implementation based on a board computer and an USB audio board may be more appropriate. In the following, in discussing the system and its actual implementation, we made the choice to focus on a compact implementation built around a PI 3 Raspberry board computer, as compact sizes and portability could be key features in a few FES applications. We will first analyze the overall structure of the system; then, we will discuss the implementation of the front-end, and finally, we will discuss the software implementation based on QLSA.

### 3.1. System Structure

**Figure 3:**
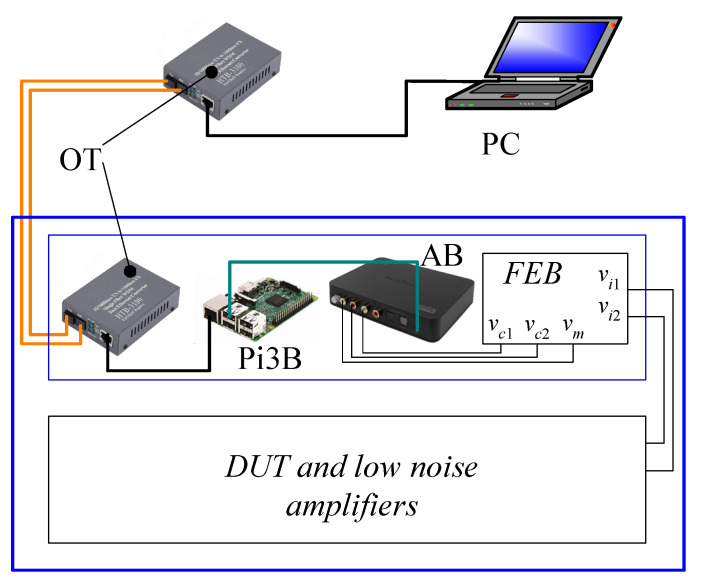
Block diagram of the implementation of the system according to the proposed approach. The square blue box represents the shielded environment in which the DUT, the low-noise electronics, and the Raspberry Pi3B based spectrum analyzer are enclosed for galvanic insulation from the external environment.

A possible system configuration in which the approach we propose can be effective is represented in Figure 3.

We assume that LFNM using two channels (for instance to allow cross-correlation measurements) must be performed on a DUT (a sensor) and that low-noise preamplifiers are available to amplify the signals of interest so that they are compatible with the input range of the Front-End Board (FEB) that performs the signal elaboration discussed in Figure 1.

The FEB receives the carrier signal from the Audio Board (AB), which in our case is an USB Sound Blaser Xi-Fi HD by Creative (maximum sampling frequency 96 kHz, resolution 24 bits). The AB receives the output signals *v_c_*_1_ and *v_c_*_2_ from the FEB. The AB is powered and controlled by a Raspberry Pi3 board computer that provides for carrier generation, signal sampling, and spectra and cross spectra estimation. The external personal computer (PC) is used for system configuration (setting carrier frequency, carrier amplitude, and spectral analysis parameters) via a client server protocol. The two Optical Transceivers (OT) are used to obtain the complete galvanic insulation between the shielded environment, into which the DUT, the low-noise amplifiers and the spectrum analyzer are located, and the external environment. The Raspberry Pi3B, the OT inside the shielded environment, and the FEB are all supplied by a standard 5 V power bank (not shown in Figure 3). In this configuration, a 10 Ah power bank can ensure continuous operation for more than 5 h. If a moderate amount of external interferences can be tolerated, the OT can be removed, and the PC and the Raspberry Pi3B can be connected directly using an ethernet cable terminated with an RJ45 connector.

### 3.2. Front-End Board

**Figure 4:**
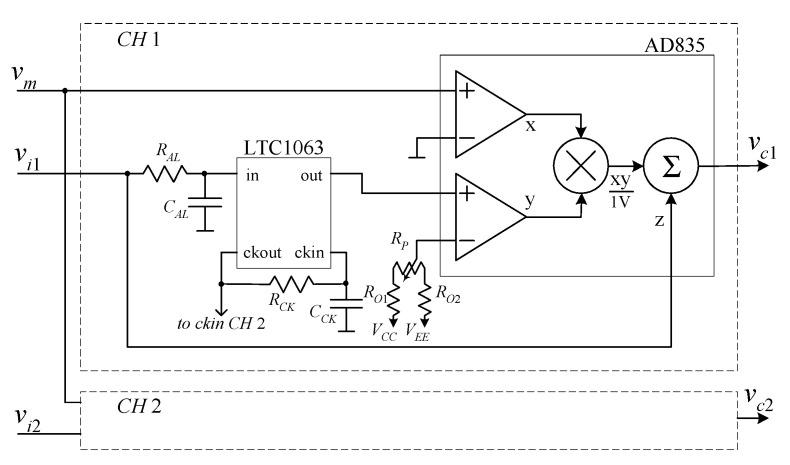
Schematic of the front-end board. *R_AL_* and *C_AL_* form an anti-aliasing low-pass filter for the 5th-order switched capacitor low-pass filter LTC1063. The corner frequency of the filter, which operates in self-clocking mode, is 1000 Hz. All the other operations required for the implementation of the approach we propose are performed by the analog multiplier AD835. The resistive networks *R_O_*_1_, *R_O_*_2_, and *R_P_* allow for nulling the DC offset.

The FEB is the only non-commercial system component. For this reason, in order to allow the replication of the system by other researchers, the FEB has been designed with the goal of low complexity and minimum number of components. The schematic of the FEB is reported in Figure 4. The only component missing from Figure 3 is the miniaturized 1 W DC-DC converter (TRACO TMA 0505D) used to obtain a dual 5 V supply (*V_CC_* = −*V_EE_* = 5 V) from the same 5 V power bank supplying the entire system. The low-pass filtered signal *v_lf_*_1_ in Figure 1 is obtained by a 5th order switched capacitor filter LTC1063. The switched capacitor filter is configured for self-clocking operation (*R_CK_* and *C_CK_*) with a clock frequency of 100 kHz, resulting in a −3 dB bandwidth of 1 kHz. A simple continuous time anti-aliasing filter (*R_AL_* = 100 kΩ *C_AL_* = 180 pF) with a corner frequency of 9 kHz is in front of the input of the switched capacitor filter.

All other required operations for signal elaboration prior to sampling are performed by the AD835 analog multiplier. The trimmer *R_P_* (*R_P_* = 1 kΩ), together with the resistances *R_O_*_1_ and *R_O_*_2_ (*R_O_*_1_ = *R_O_*_2_ = 18 kΩ) is used to allow the compensation of a DC offset coming from the low-noise amplifier that would result in a large sinusoidal component at the carrier frequency after multiplication. The correction range for the offset in our implementation is about ±130 mV, but this range can be changed by changing the value of the resistances. The second channel is almost identical to the first channel, with the only exception that the LTC1063 is not in self clocking configuration, but it receives the clock from the clock output of the LTC1063 in *CH*1 (pin ckout). The procedure used for offset correction will be discussed in the experimental section.

### 3.3. System Software

All operations for spectral estimation are performed by the Raspberry Pi3B board and are based on QLSA [23]. Communication with external PC for measurement configuration and for periodically obtaining the estimated spectra is implemented by sharing a folder between the Raspberry Pi3 and the external computer. A simple client server protocol is used based on writing and reading files on the shared folder. Both the Raspberry system software and the specification of the communication protocol are freely available to anyone interested in using the approach we propose. In this way, provided that the protocol is followed, anyone can develop his own software for system control and data representation, so that it can be tailored toward the specific measurement application one is interested in. For testing the system, we developed GUI-based application by using both MATLAB and LabWindows CVI. Typically, the user initially sets all the parameters for spectrum analysis and the frequency and amplitude of the carrier frequency. QLSA is used for both estimating the spectra over the entire frequency range and for NBSE near to the carrier frequency. Although the user can select different parameters for narrow spectra estimation, in our experiments, we selected the frequency interval between the carrier frequency *f_m_* and *f_m_* + 100 Hz. Extending the narrow spectral estimation range up to 100 Hz allows obtaining the estimation of the spectra in a frequency range (*f* > 10 Hz), which is already covered by the conventional spectral estimation process. This allows checking that the process of obtaining the low-frequency noise from the modulated signal, which involves subtracting the noise that is present at the modulating frequency and accounting for the carrier amplitude, is correct. As it is discussed in [24], the resolution in NBSE is given by the selected frequency interval divided by the record length *N* chosen upon QLSA initialization. With a typical value of *N* = 4096 and with the choice of a 100 Hz bandwidth, the frequency resolution is 24.4 mHz. However, in the case of NBSE as performed by QLSA, the *NBW* is not related to the frequency resolution. As a matter of fact, QLSA performs NBSE with a number of *NBW*s settings (in parallel) so that the user can select, at run time, the best compromise in terms of reduced systematic error due to spectral leakage and measurement time. By default, in NBSE, *N_B_* = 12 different values of NBWs are used with the bandwidth *B_i_* of the *i*th value given by:

(4) $$B_{i}=\frac{f_{S}}{N2^{i}}\;0\leq i<N_{B}.$$

With a sampling frequency *f_S_* = 48 kHz and a record length *N* = 4096, the *B_i_* ranges from about 12 Hz down to about 6 mHz. As discussed in [21], in order to reduce the systematic errors in spectral estimation due to spectral leakage, which are especially relevant when estimating flicker noise, the NBW should be well below the minimum frequency of interest. Since typical sampling frequencies for audio boards can reach 192 kHz, *N_B_* = 12 is sufficient for most situations. Note that the presence of a residual DC component can introduce a large error in the estimation of the spectra at very low frequencies. This is why it is important to correct the offset by acting on the trimmer *R_P_* in Figure 4 before performing actual measurements. The procedure for offset correction is discussed in the next subsection.

### 3.4. Offset Correction

The presence of a DC offset at the input of the multiplier results in a deterministic sinusoidal wave at the carrier frequency superimposed to the noise signal. This in turn must be avoided not only because it may result in the saturation of the inputs of the audio board but because it can cause large systematic errors in the estimation of the spectrum at very low frequencies. To understand why, it is worth noting that the process of estimation of the lower frequency portion of the spectrum performed by the NBSE engine of QLSA is, in principle, equivalent to performing a coherent demodulation of the modulated signal, followed by conventional DFT spectral estimation. A large carrier would result, after demodulation, in a large DC component that, because of spectral leakage, can result in large errors at very low frequencies [21]. While it is true that the errors introduced by a large DC component can be reduced by selecting lower and lower resolution bandwidths for spectral estimation, this would also result in longer and longer average times for obtaining a smooth spectrum. Therefore, in order not to significantly increase the measurement time, it is necessary to minimize the DC offset. Although there are many approaches that can be followed to adjust the position of *R_P_* for obtaining this result, it is generally sufficient to take advantage of the time record reporting feature incorporated within QLSA. In a preliminary measurement session, before the actual measurement is started, the presence of a large positive or negative DC offset results in a recoded time signal with a large peak-to-peak amplitude. By acting on the trimmer *R_P_*, one simply needs to reach the condition of minimum peak-to-peak amplitude, corresponding to the situation in which the carrier amplitude is much smaller than the peak-to-peak amplitude of the noise.

## 4. System Testing

**Figure 5:**
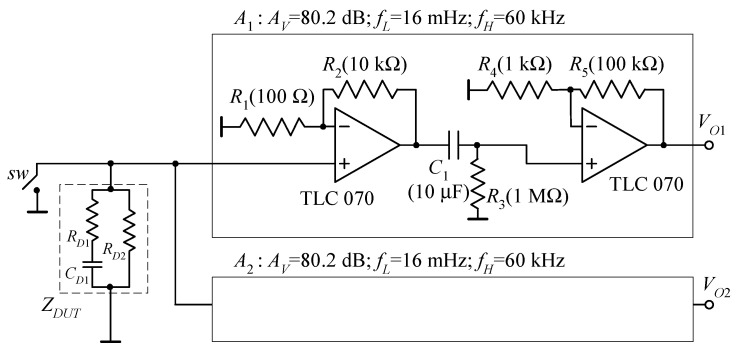
Dual channel amplifier used for experimentation. When the switch *SW* is closed, we can measure the background noise of the amplifier. With the switch open, *Z_DUT_* is used as a DUT for verifying the ability of the system to perform cross-correlation measurement in the entire frequency band of interest.

In order to validate the proposed approach, we designed two nominally identical voltage amplifiers to be used as preamplifiers for noise measurement on actual devices. The configuration with two identical amplifiers also allows performing cross-correlation measurements. The amplifiers used for the experiments are shown in Figure 5, together with the DUT used for actual noise measurement tests.

The equivalent input voltage noise of the amplifiers is rather large if compared to the best amplifiers that can be used in voltage noise measurement applications [26,27,28]. However, this is not relevant in our case, since we only want to demonstrate the ability of our system to provide the correct noise spectra down to frequencies below 1 Hz, also in cross-correlation measurements. Indeed, in this respect, a non-negligible level of equivalent input noise is even useful in demonstrating the performances that can be obtained using the cross-correlation approach. To this end, the test DUT in Figure 5 was devised in such a way that the generated noise is comparable to the equivalent input noise of each channel in Figure 5 both at high and low frequencies.

**Figure 6:**
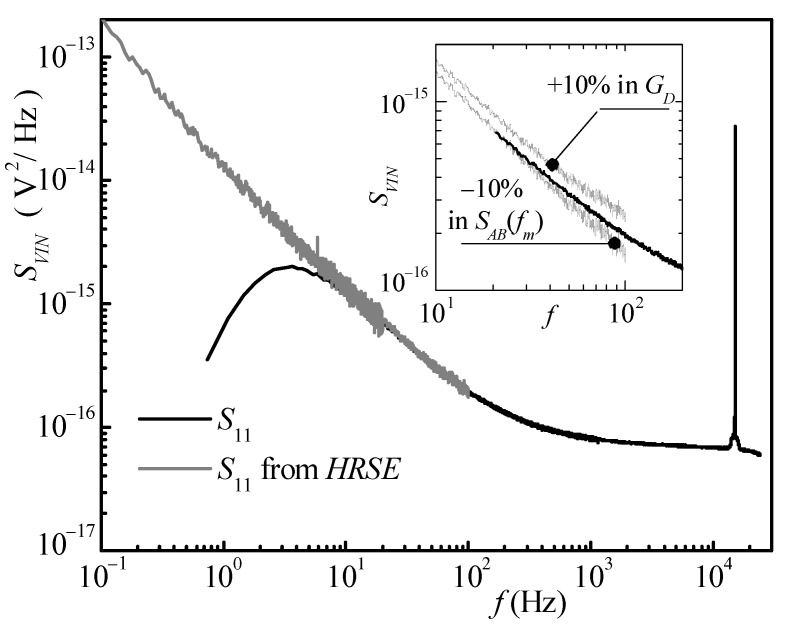
Result of noise measurements with the switch *S_W_* in Figure 5 is closed. The equivalent input noise *S_VIN_* is obtained by dividing the output noise by the amplifier gain squared. The black curve is obtained from conventional spectral estimation. The gray portion of the curve is obtained from the NBSE mode in QLSA using Equation (2). The inset shows when the incorrect values for the parameters *S_AB_*(*f_m_*) and *G_D_* in Equation (2) are chosen.

In the first measurement, we shortened the switch *SW* in Figure 5 in order to estimate the BN of the amplifiers. The result of the measurement is reported in Figure 6.

The black curve in the main graph is the PSD as estimated by QLSA in the main mode (not the NBSE mode). The effect of the audio board input high-pass filter is clearly noticeable below 10 Hz. The gray curve is the spectral estimation as obtained from the NBSE mode (in the bandwidth from *f_m_* to *f_m_* + 100 Hz) of QLSA to which Equation (2) is applied. It can be noticed that the gray curve is actually obtained by plotting curves corresponding to different NBWs. As it was discussed before, using larger NBW allows reducing the statistical error in spectral estimation in a shorter time. This results in a “smoother” curve for the same measurement time with respect to a smaller NBW. At the same time, a larger NBW may result in larger systematic errors because of window leakage at lower and lower frequencies. Therefore, as the frequency decreases, we employ the estimation coming from narrower NBWs to ensure low systematic error at the cost of larger residual statistical errors along the curve. The total measurement time was 2 h, which is a reasonable time when smooth spectra are required at frequencies as low as 100 mHz. As it can be noticed from Figure 6, and as it was expected, the estimation obtained with the conventional approach and the one based on NBSE superimpose in the interval between about 20 and 100 Hz. The fact that such a superposition should occur can actually be used for setting the correct values of *G_D_* and *S_AB_*(*f_m_*). Indeed, when even small errors in the choice of these two values are made, the superposition no longer occurs, as shown in the inset in Figure 6, where we show the curve obtained from NBSE when the correct value for *G_D_* is used and an error of −10% is made in the estimated of *S_AB_*(*f_m_*) (lower gray curve) and when the correct value of *S_AB_*(*f_m_*) is used but an error of +10% is made in the estimation of *G_D_* (upper gray curve).

To prove the ability of the proposed system to perform actual noise measurements well below 1 Hz when using the cross-correlation approach, we used the impedance *Z_DUT_* in Figure 5 as a DUT. As mentioned before, we selected the value of the resistances and of the capacitor, so that the noise generated by the DUT is comparable to the equivalent input noise of the amplifiers in Figure 5 both at high and low frequencies (*R_D_*_1_ = 1 MΩ, *R_D_*_2_ = 5.6 kΩ, *C_D_*_1_ = 6.8 nF).

**Figure 7:**
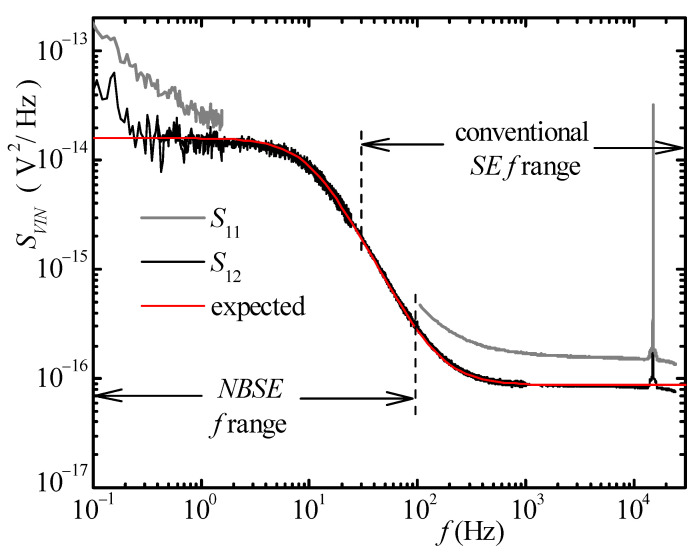
Result of noise measurements with the switch *SW* open in Figure 5. The red curve represents the expected result (nominal voltage noise across *Z_DUT_*). The gray curves represent the portion of the equivalent noise input as would be measured with a single amplifier (*CH*1). The black curve is the noise obtained from the cross-correlation between the two amplifiers. The frequency regions where spectra have been obtained from conventional Spectral Estimation (SE) and from NBSE are shown in the figure to evidence the frequency region in which the two results superimpose. The deviation with respect to the nominal value that can be observed above 10 kHz is due to the bandwidth limitation of the amplifiers in Figure 5. The deviation at frequency below 200 mHz is due to the fact that the measurement time was not sufficient to cause the complete rejection of the uncorrelated noise at these very low frequencies.

The results are shown in Figure 7. The red curve in Figure 7 represents the expected noise from the DUT in Figure 5.

The gray curve is the equivalent input noise estimated starting from the output of *CH*1 alone and is the sum of the noise from the DUT and the equivalent input noise of the amplifier. The gray curve is only shown below 2 Hz and above 100 Hz where the equivalent input noise of the amplifier is comparable or larger than the DUT noise. The black curve represents the DUT noise estimated from cross-correlation. The frequency ranges where conventional spectral estimation and estimation starting from the *NBSE* were used are indicated in the figure and, as before, there is a frequency interval (from 30 up to 100 Hz) in which the two estimates coincide. The measurement time was 3 h, and it can be noted that wherever the averaging time was sufficient to cancel the uncorrelated noise (above about 200 mHz), the result of the measurement essentially coincides with the expected PSD. Notice that the decrease in the PSD close to the carrier frequency is the result of the gain reduction of the amplifiers due to their bandwidth limits. Figure 7 clearly proves the effectiveness of the approach we propose. In actual LFNM, very low-noise amplifiers are typically used instead of the simple test amplifiers used in Figure 5. Starting from amplifiers with very low noise level at low frequency is extremely important in LFNM application since, as it is well known, and as it is clearly shown in Figure 7, while cross-correlation can be effective above a few Hz, the time required to obtain the cancellation of the uncorrelated noise at very low frequencies (<1 Hz) can be extremely long [29].

## 5. Conclusions

We have proposed and demonstrated the feasibility of a new approach for performing LFNM using standard audio boards. The only required non-off-the-shelf component is a simple front-end board used to translate very low frequencies, which would be rejected by the audio board AC input filter, at higher frequencies. The software required for the operation of the system is heavily based on the QLSA library. In this paper, we presented an implementation based on a Raspberry Pi3B board computer to meet the conditions of system compact sizes and portability, which is often required for FES applications. A copy of the file system for the Raspberry Pi3B computer and the specifications for the client server protocol to manage the system from an external PC are freely available to anyone interested in the utilization of the system. The test measurements performed on the prototype of the system demonstrated the effectiveness of the proposed approach.

## Data Availability

The data reported in the manuscript can be requested directly from the authors.
